# Progression of the Radiologic Severity Index predicts mortality in patients with parainfluenza virus-associated lower respiratory infections

**DOI:** 10.1371/journal.pone.0197418

**Published:** 2018-05-17

**Authors:** Ajay Sheshadri, Dimpy P. Shah, Myrna Godoy, Jeremy J. Erasmus, Juhee Song, Liang Li, Scott E. Evans, Roy F. Chemaly, Burton F. Dickey, David E. Ost

**Affiliations:** 1 Department of Pulmonary Medicine, The University of Texas MD Anderson Cancer Center, Houston, Texas, United States of America; 2 Department of Infectious Diseases, Infection Control and Employee Health, The University of Texas MD Anderson Cancer Center, Houston, Texas, United States of America; 3 Department of Diagnostic Radiology, The University of Texas MD Anderson Cancer Center, Houston, Texas, United States of America; 4 Department of Biostatistics, The University of Texas MD Anderson Cancer Center, Houston, Texas, United States of America; St. Jude Children’s Research Hospital, UNITED STATES

## Abstract

**Background:**

Radiologic severity may predict adverse outcomes after lower respiratory tract infection (LRI). However, few studies have quantified radiologic severity of LRIs. We sought to evaluate whether a semi-quantitative scoring tool, the Radiologic Severity Index (RSI), predicted mortality after parainfluenza virus (PIV)-associated LRI.

**Methods:**

We conducted a retrospective review of consecutively-enrolled adult patients with hematologic malignancy or hematopoietic stem cell transplantation and with PIV detected in nasal wash who subsequently developed radiologically-confirmed LRI. We measured RSI (range 0–72) in each chest radiograph during the first 30 days after LRI diagnosis. We used extended Cox proportional hazards models to identify factors associated with mortality after onset of LRI with all-cause mortality as our failure event.

**Results:**

After adjustment for patient characteristics, each 1-point increase in RSI was associated with an increased hazard of death (HR 1.13, 95% confidence interval [CI] 1.05–1.21, p = 0.0008). Baseline RSI was not predictive of death, but both peak RSI and the change from baseline to peak RSI (delta-RSI) predicted mortality (odds ratio for mortality, peak: 1.11 [95%CI 1.04–1.18], delta-RSI: 1.14 [95%CI 1.06–1.22]). A delta-RSI of ≥19.5 was 89% sensitive and 91% specific in predicting 30-day mortality.

**Conclusions:**

We conclude that the RSI offers precise, informative and reliable assessments of LRI severity. Progression of RSI predicts 30-day mortality after LRI, but baseline RSI does not. Our results were derived from a cohort of patients with PIV-associated LRI, but can be applied in validated in other populations of patients with LRI.

## Introduction

The use of mortality as a primary endpoint in clinical trials of antimicrobials has been hotly debated due to difficulty in the determination of attribution [1, 2]. The FDA recommended in 2009 that investigators consider clinical response or failure as an endpoint in trials of pneumonia [3]. Radiologic progression is considered a component of clinical failure [4, 5]. However, while some studies of pneumonia have found that radiologic progression or delayed radiologic resolution may be associated with adverse outcomes [6], other studies have failed to demonstrate this relationship [7, 8]. In other words, equipoise exists as to whether radiologic progression is clinically meaningful. The use of radiologic progression in clinical trials is limited by the fact that qualitative chest radiographic interpretations do not precisely capture degrees of change in radiologic severity [9, 10]. Moreover, qualitative chest radiograph interpretations have significant inter-observer variability (i.e., low reliability) [11, 12]. This lack of precision and reliability reduce statistical power and thereby reduce the effectiveness of qualitative interpretations of radiologic severity as an outcome measure in pneumonia trials. Currently, no validated scoring tool exists for assessing severity of radiographic infiltrates in pneumonia.

Systematic quantification of radiological severity could allow for a valid and reliable score that can serve as a biomarker of mortality. While thoracic computed tomography (CT) and chest X-ray (CXR) are important clinical tools for evaluating pneumonia [13], few studies have sought to quantify pneumonia severity radiologically [14–16]. Precise, quantitative image-based assessments of pneumonia may add prognostic value to existing clinical risk assessment tools [17], and changes in radiologic severity may predict adverse outcomes after pneumonia [6]. The development of a validated quantitative assessment of radiological severity would allow investigators to measure pneumonia progression or resolution with greater precision and reliability than a dichotomous assessment (e.g. “worsening” vs. “stable”), which would improve power. Such a tool would be well suited for inclusion as part of a composite endpoint of clinical treatment failure along with mortality.

To explore the value of quantitative assessments of radiologic severity, we sought to determine whether radiologic progression of pneumonia as quantified by a systematic scoring tool, the Radiologic Severity Index (RSI) [14], would predict mortality after lower respiratory tract infection (LRI). We tested this tool in a well-characterized cohort of patients enrolled early in the course of parainfluenza virus (PIV)-associated LRI, an infection in which mortality can approach 55% in patients with hematologic malignancies [18–20]. We further hypothesized that this scoring tool would have high reliability between readers. The primary objective of this study was to determine whether progression of LRI, as quantified by RSI, in a high-risk population of patients with LRI could predict mortality.

## Methods

### Patients

We conducted a retrospective chart review of a previously-established cohort of consecutively-enrolled adult patients with hematologic malignancies or those who had undergone hematopoietic stem cell transplantation (HSCT) who developed symptomatic PIV upper respiratory tract infection (URI) between October 1, 2002 and November 30, 2007 [18]. PIV was detected by direct immunofluorescence assay (DFA) or shell vial culture from nasal wash. We included patients with PIV URI who presented with or subsequently developed radiologic infiltrates within 28 days of PIV diagnosis in nasal wash. We excluded by consensus (A.S., D.O.) those with clinical volume overload as assessed by echocardiography, right heart catheterization, or response to diuretics, and those who had resolving infiltrates from a known prior LRI at the time of PIV detection in nasal wash. Our final cohort consisted of patients with newly-developed LRI preceded by PIV URI. This study was approved by our Institutional Review Board 4 (Federalwide Assurance #00000363) in accordance with the Helsinki Declaration of the World Medical Association with a waiver of informed consent (PA15-0892).

### Definitions

PIV-associated LRI was defined as the detection of PIV from nasal wash with symptoms of a clinical pneumonia syndrome (fever >38.3°C, cough, dyspnea and/or hypoxemia in addition to new or progressive pulmonary infiltrates seen on CT or CXR) in accordance with guidelines [21]. We chose a clinical definition of LRI since not all patients underwent bronchoscopy to detect PIV or bacterial superinfection in bronchoalveolar lavage (BAL) [22]. Onset of PIV-associated LRI was defined as the first detection of radiologic infiltrates on CXR or CT. Two investigators with experience in pulmonary medicine (A.S, D.O.) determined which patients had LRI.

### Data collection

All clinical and oncologic data were collected prospectively from a database of viral infections used in infection control. Data collected included demographic information (age, gender, race), underlying malignancy, cancer status (remission, relapse, or refractory), PIV detection in BAL (if performed), presence of BAL co-pathogens, cytotoxic chemotherapy, and administration of antiviral therapy with ribavirin and/or intravenous immunoglobulin therapy. For patients who underwent HSCT, we collected type of HSCT) (autologous vs. allogeneic, the presence of acute or chronic graft-versus-host disease (GVHD) prior to LRI, and corticosteroid administration within 30 days of infection.

### Radiologic scoring

We applied a semi-quantitative scoring tool for LRI severity initially developed in patients with H7N9 influenza LRI [14] to our study cohort due to its applicability to both CT and CXR images and ability to predict mortality. Per Fleischner Society guidelines [23], we defined consolidation as pulmonary infiltrates that obscure the margins of vessels and airway walls and ground glass opacities (GGOs) as pulmonary infiltrates that do not obscure bronchial and vascular margins. We assessed pulmonary infiltrates on CXR and CT scans in three zones: upper (above carina), middle (below carina, above inferior pulmonary vein), and lower (below inferior pulmonary vein) in both lungs.

**Fig 1:**
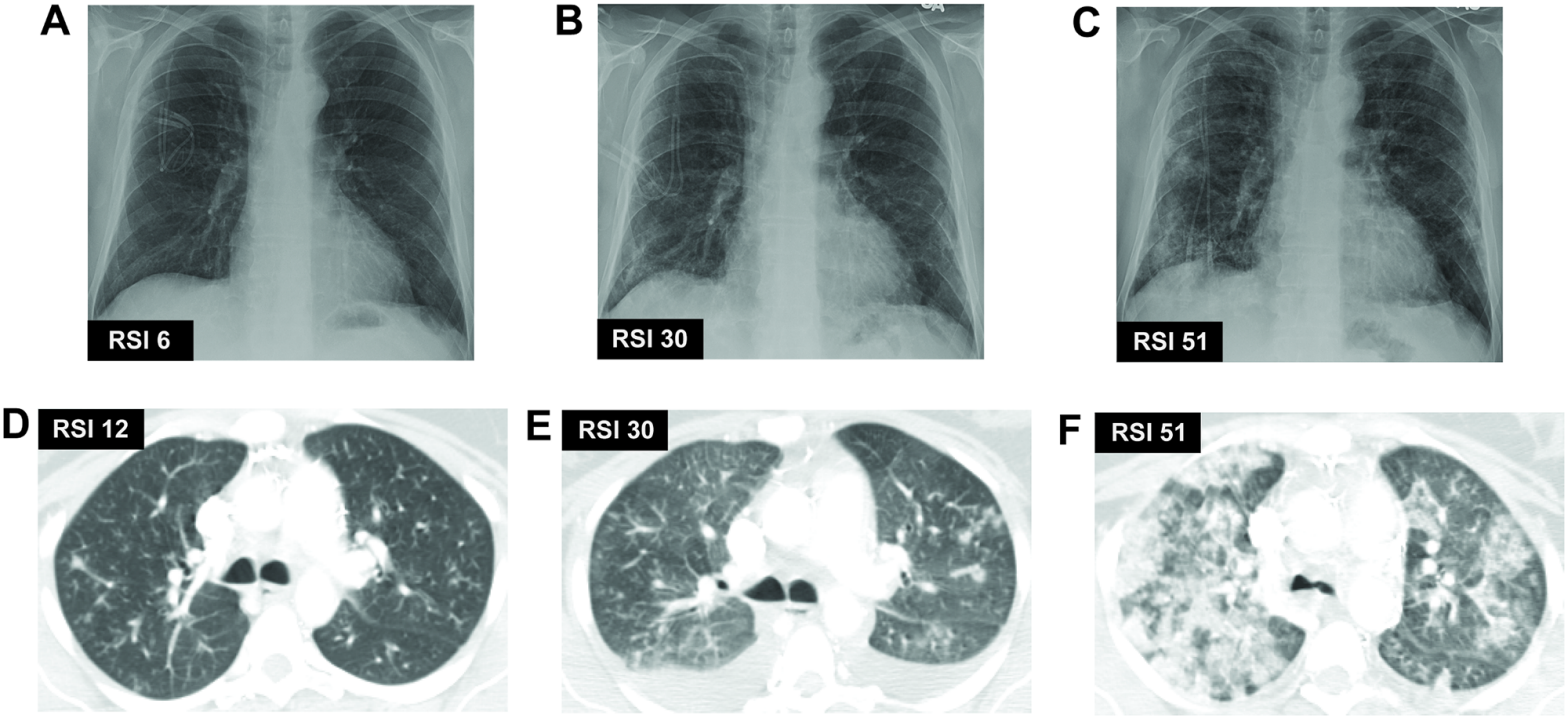
Representative images of RSI scoring for CXR and CT images. RSI scores are labelled within each panel. Panels (A)-(C) show CXR images from an individual patient in order of increasing severity. Panels (D)-(F) show CT images from a different individual patient in order of increasing severity.

Table 1 shows how RSI scores were calculated. Pulmonary infiltrates were scored on a three-point scale based on the predominant pattern in that zone: normal attenuation: 1, GGOs: 2, consolidation: 3. In general, CXR infiltrates were considered to be consolidation unless there was clear evidence of interstitial infiltrates, which were scored as GGOs. We multiplied this score by a factor based on extent of volumetric involvement: normal: 0, 1–24%: 1, 25–50%: 2, 51–75%: 3, >75%: 4. Scores from each zone were added to give the final score, called the Radiologic Severity Index (RSI), which ranges from 0–72. Dense clusters of nodules on CXR or CT were considered to be consolidative infiltrates for the purpose of RSI scoring. Volumetric assessment of the extent of infiltrates on CXR was done by the best estimate on planar anteroposterior or posteroanterior views as lateral views were not always available. Fig 1 shows representative RSI scoring for both CXR and CT images.

**Table 1:** Scoring algorithm for the Radiologic Severity Index.

Predominant Radiologic Pattern in Lung Zone	Pattern Score	Extent of Volumetric Radiologic Involvement	Volumetric Score
Normal lung	1	0% (Normal)	0
Ground glass opacities	2	1–24%	1
Consolidation	3	25–49%	2
		50–74%	3
		75–100%	4

Radiologic Severity Index (RSI) scores are calculated by multiplying the predominant pattern for each lung zone by the extent of volumetric radiologic involvement for that zone. The sum of scores from all six zones gives the final RSI, ranging from 0–72.

Two senior thoracic radiologists (M.G., J.E.) reviewed the RSI literature with two pulmonary investigators (A.S., D.O.) and scored sample CXRs and CTs from a training set to establish a consistent rule-based system for scoring images. This training set consisted of patients without PIV-associated lower respiratory tract infection (LRI) but with a broad range of severity of infiltrates. After training, the radiologists independently scored all CXR and CT images performed within 60 days of LRI in sequential order, and we used the mean of the two RSI scores for analyses. The radiologists were blinded to the primary outcome of death and to each other’s scoring. We defined baseline RSI as the RSI score on the day of LRI onset. We defined peak RSI as the highest RSI score on CXR or CT after LRI onset and delta-RSI as the change from baseline to peak RSI.

### Statistical analysis

We used chi-square or Fisher’s exact test to analyze categorical data. We used a Student’s t-test or Wilcoxon rank sum to analyze continuous data. We used an extended Cox proportional hazards model to analyze the factors associated with time to death after onset of LRI [24]. We incorporated RSI into the model as a time-varying covariate. In our primary analysis, we used RSI scores from CXR only (RSI-CXR) for the first 30 days after admission and censored patients who were alive at 30 days after LRI. In secondary analyses, we added RSI scores from CT (RSI-CT) to RSI-CXR scores in separate extended Cox models and censored all RSI scores and survival at 14, 30, or 60 days. We used the last-observation-carried-forward method to impute RSI on days in which imaging was not performed. We assumed that subjects who only had imaging performed at baseline had RSI values that remained stable throughout the study period or until they died. Variables significantly associated with mortality with p-values <0.2 in univariate analysis were candidate variables in multivariate extended Cox models, and we used backward elimination to only include variables with p<0.05. In secondary analyses, we replaced RSI with the number of involved zones (0–6). We used an extended Cox model to assess the association between the number of involved zones, expressed as a time-varying covariate, and time to death. We then compared models using RSI with models using the number of involved zones by using the generalized R^2^ method to determine which model was best [25]. Generalized R^2^ estimates in Cox models are often low due to censored data and do not reflect the robustness of models; however, this technique can compare different models.

We used univariate logistic regression models with baseline, peak and delta-RSI as separate predictors of 30-day mortality. We used Bland-Altman plots [26], paired t-tests, Pitman-Morgan tests and intra-class correlation (ICC) to assess agreement, bias, and variance between readers and diagnostic modalities using all data up to 60 days post-LRI. P-values <0.05 were considered statistically significant and all tests were two-sided. We used SAS v9.4 (SAS Institute Inc., Cary, NC) for statistical analyses.

## Results

### Baseline characteristics

**Fig 2:**
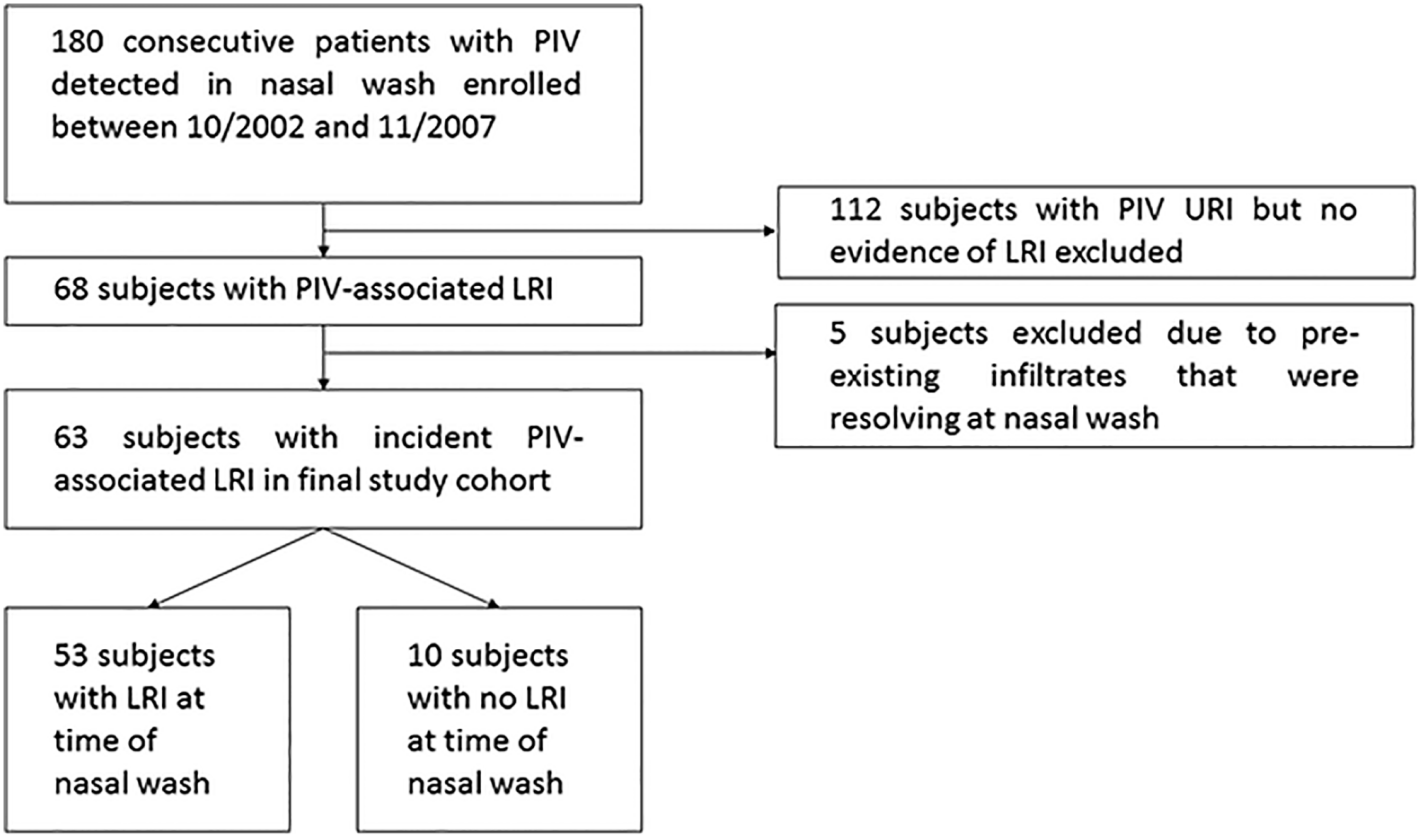
Enrollment flow chart. Enrollment flow chart for patients with parainfluenza virus (PIV)-associated lower respiratory tract infection (LRI) included in this study for analysis. BAL = bronchoalveolar lavage.

Fig 2 shows our selection of the study cohort. We excluded five patients who had resolving LRI at the time of PIV diagnosis and did not exclude any patients due to volume overload at the time of PIV diagnosis. Table 2 compares baseline characteristics between survivors and non-survivors at 30 days after LRI onset. Survivors were more likely to be female (survivors: 50%, non-survivors 11%, p = 0.04). Otherwise, there were no significant differences in age, race, underlying malignancy, remission status, or exposure to cytotoxic chemotherapy within 30 days between survivors and non-survivors. In patients who underwent bronchoscopy (n = 32), detection of PIV in BAL fluid was not significantly associated with mortality (non-survivors: 2/3, 67% detection, survivors: 6/29, 21% detection, p = 0.15), possibly due to the small number of patients who underwent BAL. Only two patients had co-infection with other pathogens detected in BAL fluid; cytomegalovirus was detected in one patient, and herpes simplex virus in the other. There was no significant difference in mortality between HSCT and non-HSCT patients (HSCT: 11.5%, non-HSCT: 16.2%, p = 0.60). Table 3 shows baseline characteristics for the subgroup of patients who had undergone HSCT. No significant differences existed between survivors and non-survivors in time from HSCT to LRI, HSCT type (autologous vs. allogeneic), transplant source (bone marrow, cord blood, or peripheral blood), administration of antiviral therapy, or baseline corticosteroid dose.

**Table 2:** Baseline characteristics by 30-day survival status.

Variable	Survivors (n = 54)	Non-Survivors (n = 9)	p-value^a^
Age (years, mean±SD)	52±15	59±14	0.17
Gender (% female)	27(50%)	1(11.1%)	0.04
*Race (n*,*%)*			
Non-Hispanic White	39(72.2%)	8(88.9%)	1.00
Asian	4(7.4%)	1 (11.1%)	
Black	5(9.3%)	0	
Hispanic	5(9.3%)	0	
Middle Eastern	1(1.9%)	0	
*Underlying malignancy (n*,*%)*			0.10
Acute lymphocytic leukemia	13 (24%)	0 (0.0%)	
Acute myelogenous leukemia	14 (26%)	7 (77.8%)	
Chronic lymphocytic leukemia	4 (7.4%)	1 (11.1%)	
Chronic myelogenous leukemia	7 (13.0%)	0 (0.0%)	
Myelodysplastic syndrome	1 (1.9%)	0 (0.0%)	
Hodgkin’s lymphoma	1 (1.9%)	1 (11.1%)	
Non-Hodgkin’s lymphoma	8 (14.8%)	0 (0.0%)	
Multiple myeloma	3 (5.6%)	0 (0.0%)	
Ovarian cancer	2 (3.7%)	0 (0.0%)	
T-cell prolymphocytic leukemia	1 (1.9%)	0 (0.0%)	
*Cancer status at time of LRI (n*,*%)*^b^			0.38
Active	14(25.9%)	1(11.1%)	
Remission	8(14.8%)	0 (0.0%)	
Refractory	20(37%)	4(44.4%)	
Relapse	12(22.2%)	4(44.4%)	
Cytotoxic chemotherapy within 30 Days (n,%)	34(64.2%)	6(75%)	0.70

Lower respiratory tract infection, LRI

^**a**^ Fisher’s exact test for categorical data and Student’s t-test for continuous data

^b^ Active = undergoing initial treatment for cancer; remission = disease-free at the time of enrollment for at least 6 months; relapse = disease occurring after remission; refractory = not receiving initial treatment for cancer and never having achieved remission.

**Table 3:** Characteristics of patients with hematopoietic stem cell transplants.

Variable	Survivors (n = 23)	Non-survivors (n = 3)	p-value^a^
Days from HSCT to LRI (median, interquartile range)	134 (21–790)	171 (131–335)	0.81^b^
*Type of transplant (n*,*%)*			0.22
Autologous	6(26.1%)	2(66.7%)	
Allogeneic	17(73.9%)	1(33.3%)	
*Source of transplant (n*,*%)*			1.00
Bone marrow	11(47.8%)	2(66.7%)	
Umbilical cord blood	1(4.3%)	0	
Peripheral blood	11(47.8%)	1(33.3%)	
*Corticosteroid dose (n*,*%)*			0.77
None	9(39.1%)	1(33.3%)	
Low dose	6(26.1%)	0	
High Dose	8(34.8%)	2(66.7%)	

^**a**^ Fisher’s exact test for categorical data and Student’s t-test for continuous data

^b^ Wilcoxon rank-sum test

HSCT = hematopoietic stem cell transplantation; LRI = lower respiratory tract infection; corticosteroid low dose, <600 mg/week of prednisone or equivalent at baseline; corticosteroid high dose = ≥600 mg/week of prednisone or equivalent at baseline.

### Change in Radiologic Severity Index as a predictor of mortality

**Fig 3:**
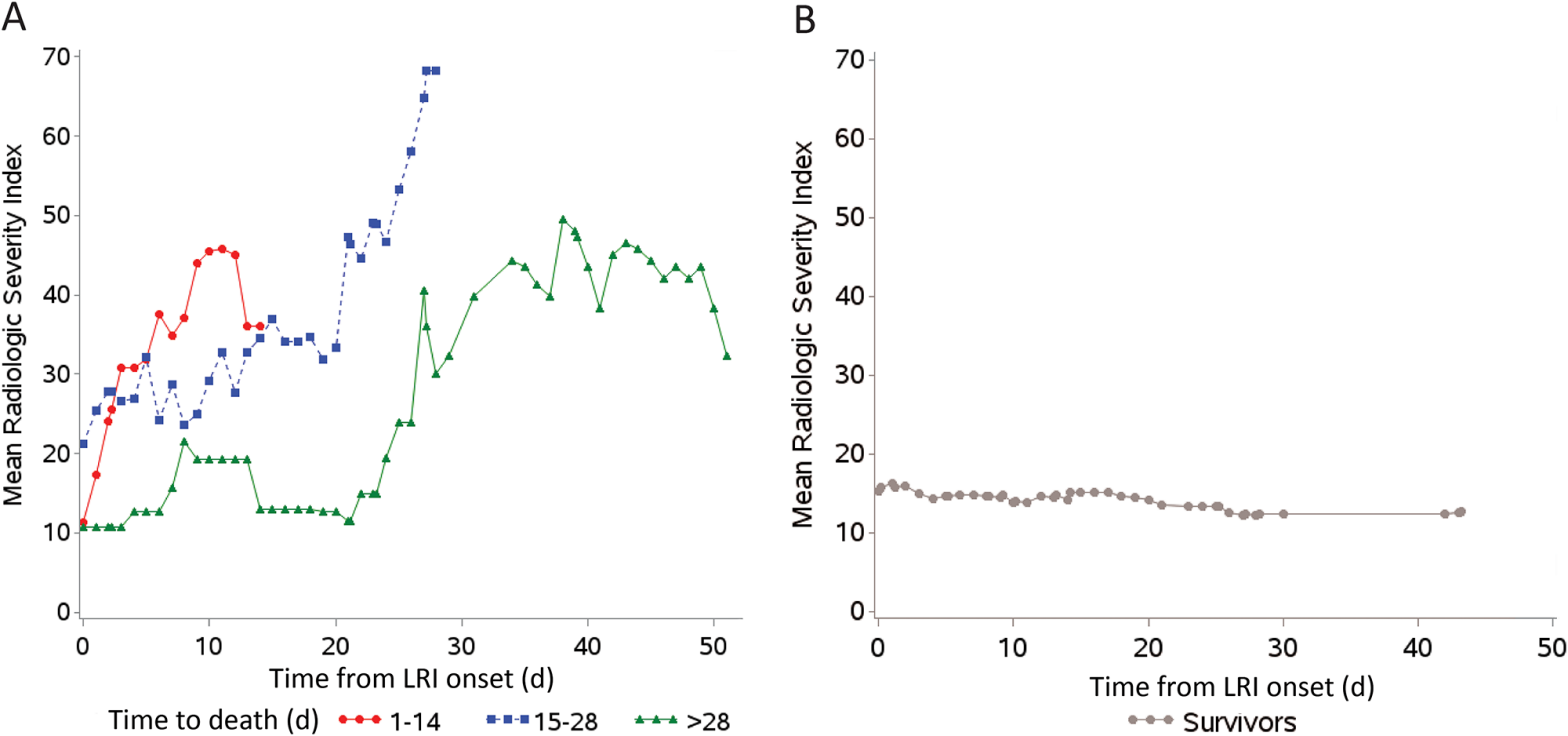
Trends in mean Radiologic Severity Index (RSI) scores over time in non-survivors (A) and survivors (B). (A) Trends in mean RSI scores are shown for patients who died within 14 days (solid circles, n = 4), patients who died between days 15 and 28 (solid squares, n = 5), and patients who died after day 28 (solid triangles, n = 2). (B) Trends in mean RSI scores are shown for survivors.

In our primary extended Cox model, we incorporated 241 measurable RSI-CXR scores. Non-survivors underwent more radiologic tests within the study period (median number of total radiologic tests—non-survivors: 6, survivors: 3). In univariate Cox regression models, each 1-point increase in RSI-CXR measured as a time-varying covariate was associated with a 13% increase in the hazard for death (HR 1.13, 95% confidence interval [CI] 1.06–1.19, p<0.0001). The proportional hazards assumption was met. AML was associated with mortality in our univariate Cox regression model (HR 8.1, 95%CI 1.7–39.1, p = 0.01). After adjusting for age, presence of AML and gender, only the progression of RSI remained in the multivariate model and predicted mortality (multivariate HR 1.13, 95%CI 1.05–1.21, p = 0.0008, Table 4). Fig 3 shows longitudinal changes in mean RSI between survivors and non-survivors. Non-survivors had increases in RSI temporally associated with time of death, whereas RSI remained stable in survivors.

**Table 4:** Univariate and multivariate cox regression models for the prediction of 30-day mortality.

Variable	Univariate HR (95% CI, p-value)	Multivariate HR (95% CI, p-value)
Age	1.03 (0.98–1.09, p = 0.19)	
*Gender*		
Female	1.0	
Male	7.5 (0.9–59.6, p = 0.06)	
*Race*		
Non-White	1.0	
White	2.9 (0.4–22.9, p = 0.32)	
*Underlying Malignancy*		
Non-AML	1.0	
AML	8.1 (1.7–39.1, p = 0.01)	
*Cancer status at time of LRI*^a^		
Active	1.0	
Remission	0.0 (p = 0.99)	
Refractory	2.4 (0.3–21.7, p = 0.43)	
Relapse	3.9 (0.4–35.1, p = 0.22)	
*Cytotoxic Chemotherapy*		
No	1.0	
Yes	1.6 (0.3–8.1, p = 0.55)	
RSI^b^	1.13 (1.06–1.19, p<0.0001)	1.13 (1.05–1.21, p = 0.0008)

AML: acute myelogenous leukemia; CI: confidence interval; RSI: radiologic severity index

^a^ Active = undergoing initial treatment for cancer; remission = disease-free at the time of enrollment for at least 6 months; relapse = disease occurring after remission; refractory = not receiving initial treatment for cancer and never having achieved remission

^b^ RSI was used as a time-varying covariate in both models. The remaining variables were entered into the model with only baseline values.

Abbreviations: AML = acute myelogenous leukemia; RSI = Radiologic Severity Index

In separate univariate Cox models using all RSI-CXR and RSI-CT scores for 14-, 30- or 60-day outcomes, RSI remained predictive of mortality (14-day mortality: HR 1.13, 95%CI 1.04–1.22, p = 0.005; 30-day mortality: HR 1.12, 95% CI 1.05–1.20, p = 0.0005; 60-day mortality: HR 1.11, 95%CI 1.06–1.17, p<0.0001). In secondary univariate analyses, extended Cox models using the number of involved zones predicted mortality (HR 2.24 per involved lobe, 95%CI 1.17–4.30, p = 0.02), but RSI was better at predicting mortality than the number of involved zones (RSI: R^2^ = 0.11, number of zones: R^2^ = 0.04) [25].

### Prediction of mortality using baseline, peak and delta-RSI

Using univariate logistic regression models, we found that longitudinal assessments of RSI were informative, whereas baseline assessments were not. Mean delta-RSI scores were highly predictive of mortality (non-survivors: 33.6±5.6, survivors: 5.6±33.6, p<0.0001), as were peak RSI scores (non-survivors: 53.2±13.2, survivors: 18.9±17.6, p<0.0001) (Table 5). However, baseline RSI scores were not predictive of mortality (non-survivors: 19.6±8.8, survivors: 13.3±14.3, p = 0.22). Adding RSI-CT scores to the logistic regression models improved the predictive power of delta-RSI but not peak RSI (Table 6). Delta-RSI using only RSI-CXR scores had good discrimination (area under the ROC curve: 0.92) but this discrimination improved with the addition of RSI-CT scores (area under the ROC curve: 0.97).

**Table 5:** Univariate logistic regression models for the prediction of 30-day mortality with baseline RSI, peak RSI and delta-RSI with RSI-CXR scores.

Predictor	Odds Ratio (95% CI)	p-value	AUC	Cutoff value ^a^	Sensitivity	Specificity
Baseline RSI	1.029 (0.983–1.077)	0.2170	0.74	12	100%	53%
Peak RSI	1.106 (1.041–1.176)	**0.0012**	0.92	36	100%	79%
Delta-RSI	1.137 (1.061–1.219)	**0.0003**	0.89	19.5	89%	91%

One patient was excluded from this analysis due to having no serial RSI-CXR measurements

^a^ Cutoff value by maximum Jensen index

Abbreviations: RSI = Radiologic Severity Index; AUC = area under the ROC curve; ICU = intensive care unit.

**Table 6:** Univariate logistic regression models for the prediction of 30-day mortality with baseline RSI, peak RSI and delta-RSI with any RSI score.

Predictor	Odds Ratio (95% CI)	p-value	AUC	Cutoff value ^a^	Sensitivity	Specificity
Baseline RSI	0.986 (0.931–1.044)	0.6232	0.53	0	33%	87%
Peak RSI	1.111 (1.044–1.182	**0.0009**	0.92	36	100%	78%
Delta-RSI	1.150 (1.062–1.247)	**0.0006**	0.97	19.5	100%	89%

^a^ Cutoff value by maximum Jensen index

Abbreviations: RSI = Radiologic Severity Index; AUC = area under the ROC curve; ICU = intensive care unit

### Reliability of RSI between readers

**Fig 4:**
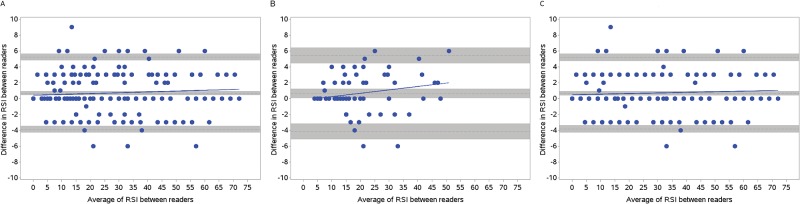
Bland-Altman plots for agreement between expert radiologists. Bland-Altman plots for agreement between expert radiologists in (A) RSI, (B) RSI scores restricted to computed tomography (CT) only (RSI-CT), and (C) RSI scores restricted to chest x-ray (CXR) measurements only (RSI-CXR). Upper and lower gray bars show 95% confidence intervals for upper and lower limits of agreement. Center gray bar shows 95% confidence intervals for bias. Solid line represents the slope of the bias (p = NS).

Reliability between readers was excellent for all RSI scores (ICC: 0.99). Table 7 shows the distribution of radiologic patterns in CT and CXR as scored by each reader. We included all available images that were scored for all patients within 60 days of LRI onset (n = 338). Fig 4 shows Bland-Altman plots for agreement between readers in RSI, RSI-CT and RSI-CXR. There was no difference in variance between readers; reader 1 systematically assigned higher RSI scores to images as compared with reader 2, but the difference was <1 unit RSI (Table 8).

**Table 7:** Distribution of radiologic patterns in chest X-rays and chest computed tomography scans.

Predominant Radiologic Pattern	Reader 1 (n,%)	Reader 2 (n,%)
		
**CXR**		
Normal Attenuation	536 (33%)	573 (35%)
Ground Glass Opacities	41 (3%)	45 (3%)
Consolidation	1043 (64%)	1002 (62%)
**CT**		
Normal Attenuation	78 (19%)	84 (21%)
Ground Glass Opacities	182 (45%)	182 (45%)
Consolidation	148 (36%)	142 (35%)

Abbreviations: CXR = chest X-ray, CT = computed tomography of the chest

**Table 8:** Agreement between readers for RSI scores.

Measure	N	Reader 1	Reader 2	Bias ^a^	Limits of agreement (95% CI) ^c^
		Mean±SD	Mean±SD	Mean±SD (95% CI)	
RSI	338	24.0±18.6	23.3±18.4	0.7±2.3 (0.4, 0.9), p<0.0001^b^	(-3.9, 5.2)
RSI-CT	68	19.72±12.1	19.1±11.6	0.6±2.4 (0.0, 1.2), p = 0.04^b^	(-4.2, 5.4)
RSI-CXR	270	25.0±19.7	24.4±19.6	0.7±2.3 (0.4, 0.9), p<0.0001^b^	(-3.8, 5.2)

Abbreviations: RSI = Radiologic Severity Index, RSI-CT = RSI scores restricted to computed tomography (CT) only, RSI-CXR = RSI scores restricted to chest X-ray (CXR) measurements only

Bland-Altman analyses for agreement between expert radiologists for RSI scores.

^a^ Bias: Reader 1 –Reader2

^b^ Pitman-Morgan test for difference in variance p = NS

^c^ 95% confidence interval (CI) for upper and lower limits of agreement in: RSI lower limit, (-4.3,-3.5); RSI upper limit, (4.8,5.6); RSI-CT lower limit, (-5.2,-3.2); RSI-CT upper limit, (4.4,6.4); RSI-CXR lower limit, (-4.3,-3.4); RSI-CXR upper limit, (4.7,5.6)

### Reliability of RSI between radiologic modalities

**Fig 5:**
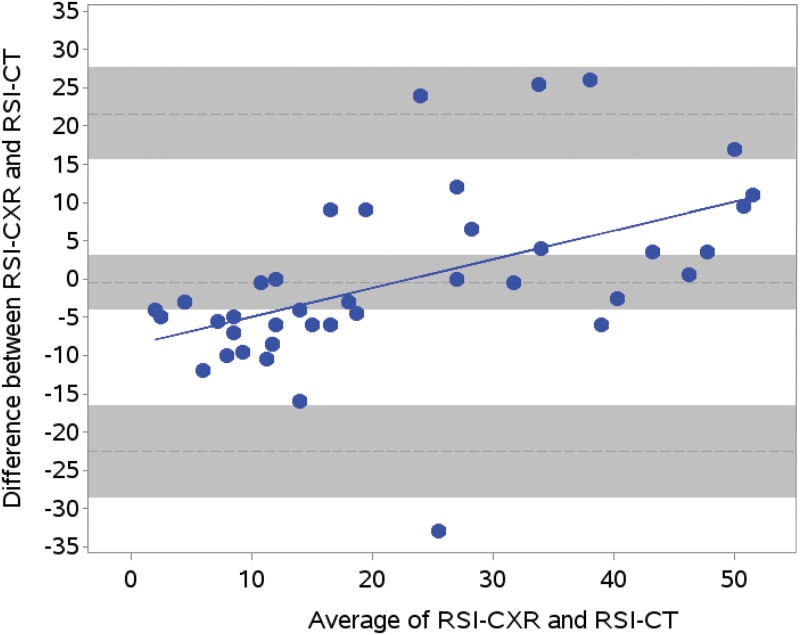
Bland-Altman plots for agreement between RSI-CXR and RSI-CT. Upper and lower gray bars show 95% confidence intervals for upper and lower limits of agreement. Center gray bar shows 95% confidence intervals for bias. Solid line represents the slope of the bias (slope = 0.38; p = 0.0008). RSI-CXR systematically underestimates RSI-CT at low RSI scores and overestimates RSI-CT at high RSI scores.

Fig 5 shows a Bland-Altman plot comparing the first available RSI-CT with the closest RSI-CXR obtained within 2 days of RSI-CT. We identified 41 CXR-CT dyads that were performed within 48 hours of each other in the same patient. The reliability between RSI-CT and RSI-CXR was good (intra-class correlation: 0.76). There was no fixed bias between RSI-CXR and RSI-CT (Table 9); however, there was bias which changed with the severity of LRI (Fig 5). When LRI was mild, RSI-CXR was systematically lower than RSI-CT (i.e. RSI-CXR underestimated RSI-CT). When LRI was severe, RSI-CXR was systematically higher than RSI-CT (i.e. RSI-CXR overestimated RSI-CT). RSI-CXR demonstrated higher variance than RSI-CT (Pitman-Morgan p = 0.0008).

**Table 9:** Agreement between RSI-CXR and RSI-CT.

Measure	N	RSI-CXR	RSI-CT	Bias ^a^	Limits of Agreement (95% CI) ^c^
		Mean±SD	Mean±SD	Mean±SD (95% CI)	
RSI	41	21.7±18.5	22.2±13.1	-0.5±11.3 (-4.0, 3.1) ^b^	(-22.5, 21.6)

Abbreviations: RSI = Radiologic Severity Index, RSI-CT = RSI scores restricted to computed tomography (CT) only, RSI-CXR = RSI scores restricted to chest X-ray (CXR) measurements only

Bland-Altman analyses for agreement between RSI-CT and RSI-CXR when both measures were obtained within 48 hours of each other.

^a^ Bias: RSI-CXR—RSI-CT

^b^ paired t-test p = NS, Pitman-Morgan test for difference in variance p = 0.0008

^c^ 95% confidence interval (CI) for upper and lower limits of agreement: lower limit, (-28.6, 16.5); upper limit, (15.6, 27.7)

## Discussion

We present the Radiologic Severity Index (RSI), a systematic radiologic scoring tool for LRI severity previously used in H1N1 influenza LRI [14], and tested its utility in patients with clinical PIV-associated LRI. Progression of RSI after adjusting for baseline factors predicted mortality. Delta-RSI and peak RSI were accurate predictors of 30-day mortality. However, baseline RSI was not predictive of mortality. RSI performed better than traditional qualitative radiologic assessments of LRI severity. A major advantage of RSI over qualitative radiologic assessments is high inter-observer reliability. We propose that the RSI is a valid and reliable tool for the assessment of radiologic severity of LRI and would serve well as a biomarker of mortality in clinical trials as part of a composite endpoint of clinical treatment failure.

Few studies have attempted to systematically quantify LRI severity radiologically. Grieser et al. developed a semi-quantitative CT-scoring tool for the acute respiratory distress syndrome after H1N1 influenza LRI in 23 patients [15]. This tool scored infiltrates (normal/GGO/consolidation) identically to RSI but estimated extent of involvement to the nearest 10% on CT instead of 25% as with RSI. A CT-score of >100 (range 0–600) at H1N1 diagnosis was associated with higher mortality and requirement for extracorporeal membrane oxygenation. Feng et al. modified this CT-score by estimating the extent of involvement to the nearest 25% and applying the score to both CXRs and CTs performed at H7N9 LRI diagnosis in 22 patients [14]. A score of >19 on CXR or >22 on CT was predictive of mortality, with CT having more predictive value. We modified Feng et al.’s simplified quantitative tool by added further scoring guidelines (enumerated in the Methods) in order to improve reliability, especially regarding the scoring of CXRs. Our modifications retain the granularity of the above iterations but allow for easier replication of RSI scoring.

Our study of RSI adds to the existing literature by showing that progression of RSI on longitudinal assessment predicts mortality after PIV-associated LRI. Moreover, delta-RSI had excellent discriminatory ability to predict 30-day mortality, while baseline RSI scores were not predictive of mortality after PIV-associated LRI. This demonstrates that it is the change in radiologic severity that is informative. Commonly used clinical risk assessment tools for community acquired pneumonia (CAP), such as the CURB-65, were primarily developed to predict outcomes using baseline data and therefore cannot serve as outcome measures of pneumonia [27–32]. In addition, these clinical risk assessment tools are only valid when measured on admission, but are not valid when used as serial longitudinal assessments. In contrast, our data show that longitudinal assessments of RSI, unlike baseline assessments of RSI, are highly associated with mortality and therefore can be used as an outcomes in trials of pneumonia.

Our findings build on those of other investigators. A substantial proportion of patients have persistent infiltrates weeks after LRI onset. Mittl et al. found that in 81 patients hospitalized with CAP, 33% had delayed resolution of pneumonia [7], but this was not associated with mortality. Bruns et al. found that in 288 patients hospitalized for CAP, 47% had infiltrates 28 days after onset of CAP and delayed resolution of CAP was associated with more severe disease [8], although progression of infiltrates was not associated with mortality. Lisboa et al. found that in 457 ICU patients, rapid progression of CAP, as defined by >50% increase in CXR infiltrates from baseline to 48 hours, was a stronger predictor of shock and mortality than bacteremia [6]. While all of these prior investigators found that radiographic changes are common during the course of LRI, their conclusions regarding the relationship between radiographic changes and mortality vary. We believe that the reason for this variability in conclusions between prior reports is due at least in part to differences in patient selection and analytic methodology (S1 Discussion). Our population was ideal for testing the validity of RSI as we focused on a well-defined population of patients who had a high baseline risk for mortality and who had early LRI, similar to Lisboa et al [6]. Mortality in pneumonia can range from 7% in non-immunocompromised inpatients with CAP [33] to >50% in immunocompromised patients with pneumonia [34–36]. By studying RSI in a population of patients with a high baseline risk of mortality after PIV-associated LRI [18–20], we ensured that we had a sufficiently high event rate to measure the effect of LRI progression on mortality. In addition, our focus on early LRI allowed us to preserve the potential for us to capture the full magnitude of worsening radiologic severity. Our use of longitudinal assessments with extended Cox models employing time-varying covariates gave us greater power to measure the effects of LRI progression on mortality due to the ability to precisely measure the progression of radiologic severity while accounting for changing severity and without introducing bias.

In addition, precise quantification of radiographic results likely improved our ability to detect associations of radiologic severity with mortality. Others have found that multilobar pneumonia, as determined on qualitative chest radiograph interpretation, is associated with death [37–41], treatment failure [5] and delayed resolution of pneumonia [42]. Our study adds to these observations by showing that mortality increases in proportion to the number of zones involved through the use of time-varying covariates. However, RSI was a more robust estimator of LRI severity, likely due to RSI’s provision of an estimate of the extent of involvement of each lobe in addition to the number of lobes involved, allowing greater precision in severity estimates. For example, the use of number of involved lobes would classify multilobar scattered GGOs as more severe than a dense unilobar consolidation. More precisely quantifying radiologic progression using RSI improves the ability to detect meaningful radiologic changes. Furthermore, using RSI as a defined outcome has the potential to improve statistical power to detect differences over conventional qualitative assessments.

The high reliability of RSI among readers improves precision in the assessment of radiologic severity. In studies using qualitative interpretations of radiologic severity, inter-observer variability between readers of chest radiographs may have impacted the ability to find an association between radiologic severity and mortality. For example, El Solh et al found that when determining whether chest radiographs had consolidation, interstitial infiltrates, or a mixed pattern, the kappa statistic between two radiologists was 0.6. [42] Taylor et al. found a kappa statistic of 0.75–0.83 between two radiologists when using a 5-point scale for pneumonia severity [16]. In comparison, we observed an ICC of 0.99 between two expert radiologists scoring RSI on a 72-point scale, which is excellent and compares favorably to the reliability reported by other investigators. We propose that the high inter-observer reliability of RSI is due to the granularity of scoring and the ability to capture the entire continuum of radiologic progression from minimal to extensive. This granularity and high reliability are necessary characteristics for imaging biomarkers [43].

Others have highlighted the discrepancy between CXR and CT when diagnosing LRI, with discordance between imaging modalities in the diagnosis of LRI in 27–48% of patients [13, 44, 45]. CXR may underestimate or overestimate the presence of LRI as compared with CT and the agreement between the two modalities is generally poor [13, 46]. Our study adds to the existing literature by showing that RSI-CXR systematically underestimates RSI-CT when infiltrates were sparse and systematically overestimates RSI-CT when infiltrates were dense. This is likely because when infiltrates are sparse, they are more likely to be detected by CT due to cross-sectional imaging, but when infiltrates are dense, CXR is more likely to over-estimate severity due to superimposition of opacities over unaffected lung. Due to the retrospective nature of our study, the temporal dissociation between CT and CXR assessments, and the relative scarcity of RSI-CT measurements, we cannot accurately estimate the gain in accuracy with the use of RSI-CT over RSI-CXR. However, our study suggests that the addition of RSI-CT scores to RSI-CXR scores improves the predictive power of RSI when using delta-RSI. Further work is necessary to define optimal imaging strategies for assessing radiologic severity.

Not all patients in this study had proven PIV LRI, which is defined as detection of PIV from a lower respiratory tract sample and has been well established as a criterion [19, 47, 48]. However, the mortality after any PIV infection remains high in patients with hematologic malignancy or HCT recipients [18]. Furthermore, bronchoscopy is not performed routinely at our institution in all patients with PIV infection detected by other means. Therefore the diagnosis of proven PIV LRI may have been biased by selection for bronchoscopy. Therefore, we chose to study RSI in a well-characterized cohort of patients with newly-developed LRI following PIV URI who had a high baseline risk for mortality after LRI.

Our study must be interpreted in the context of certain limitations. Since we tested RSI only in a cohort of patients with PIV-associated LRI, further validation is necessary in other cohorts with LRI such as CAP or RSV-associated LRI. The study was retrospective in nature, and measurements were not obtained systematically. Measurement bias may exist because sicker patients were likely to have more assessments, and patients who died may not have had radiologic assessment at peak severity. However, the lack of follow-up studies in healthier patients would likely bias our results towards the null hypothesis, since we do not capture resolution of pneumonia. Furthermore, the lack of radiologic studies in patients who died before follow-up studies could be obtained would also bias our results towards the null hypothesis, because we would miss the progression of severe pneumonia. Our method of imputation ensured that our model likely captured progression of clinical disease, since radiology is typically performed due to changes in clinical status and not performed when a patient’s clinical status is stable (S2 Discussion). Radiologic tests were scored sequentially and not in random order, which may have introduced bias if knowledge of prior scores impacts the scores of later studies. Our study sample was small, though we had adequate power due to longitudinal assessments and time-to-event analyses. Our cohort was enrolled over 10 years ago, but treatment of PIV infection is still limited to supportive care in the modern era. In addition, as in more modern cohorts, our mortality rate in this study was high, which was necessary to test the validity of RSI [19]. However, we used all-cause mortality as an endpoint instead of mortality attributed to LRI; therefore, it is possible that some patients died of unidentified causes not directly related to PIV-associated LRI. Because we detected PIV infection using DFA and not nucleic acid amplification testing (NAAT), we may have missed some patients with PIV infection. However, patients with PIV infection who are detected by NAAT but not DFA have a similar risk of death [19]. Because of these concerns, RSI should be validated in a more contemporaneous cohort. Despite exclusion of baseline volume overload, some patients may have developed volume overload after enrollment; however, this would likely bias results toward the null hypothesis if volume overload were associated with lower mortality than LRI. Because the RSI is intended to capture radiologic infiltrates that are more diffuse, it may not be appropriate to score infections that tend to present with scattered nodular infiltrates on thoracic imaging, such as fungal infections.

## Conclusions

In summary, we show that progression of radiologic infiltrates as assessed by the RSI is predictive of mortality in a high-risk cohort of patients with LRI. Longitudinal assessments of RSI were predictive of mortality, while baseline assessments were not. RSI was superior to previous methods to quantify LRI severity due to improved precision and reliability. Substantial discrepancies exist between CT and CXR assessments of LRI severity. RSI was tested in a cohort of patients with PIV-associated LRI, and future studies are needed to validate RSI in other LRI populations and to correlate RSI with other important outcomes such as health care utilization and cost. RSI is a promising tool that offers granular, reliable, precise and informative assessments of LRI severity and is well suited for consideration as part of a composite endpoint of clinical treatment failure in therapeutic trials of pneumonia.

## Supporting information

S1 Discussion Selection of study cohort when using RSI to predict mortality. This section offers a brief discussion on the utility of RSI depending upon a study population’s baseline risk of death and based on the type of statistical analysis employed.
(DOCX)

S2 Discussion Measurement bias and data sampling in retrospective and prospective cohort studies. This section offers a brief discussion on pitfalls of data sampling in retrospective and prospective cohort studies, with suggestions on optimal data sampling techniques for potential prospective studies using RSI as a biomarker of radiological pneumonia severity.
(DOCX)

S1 Dataset Deidentified dataset in Excel (.xls) format. (XLS)

## Abbreviations

BAL: Bronchoalveolar lavage
CAP: Community acquired pneumonia
CT: Computed tomography
CXR: Chest X-ray
GGOs: Ground glass opacities
HSCT: Hematopoietic stem cell transplantation
ICU: Intensive care unit
LRI: Lower respiratory tract infection
PIV: Parainfluenza virus
ROC: Receiver Operating Characteristics
RSI: Radiologic Severity Index
URI: Upper respiratory tract infection
