# Supplementary material for: Progression of the Radiologic Severity Index predicts mortality in patients with parainfluenza virus-associated lower respiratory infections
Source: PLoS One. 2018 May 17;13(5):e0197418. doi: 10.1371/journal.pone.0197418 (PMC5957350; doi:10.1371/journal.pone.0197418)
Supplement: S1 Discussion — This section offers a brief discussion on the utility of RSI depending upon a study population’s baseline risk of death and based on the type of statistical analysis employed. (DOCX) [file pone.0197418.s001.docx]

**Progression of the Radiologic Severity Index Predicts Mortality in Patients with Parainfluenza Virus-Associated Lower Respiratory Infections (Online Supplement)**

Ajay Sheshadri^1*^, Dimpy P. Shah^2#a^, Myrna Godoy^3^, Jeremy J. Erasmus^3^, Juhee Song^4^, Liang Li^4^, Scott E. Evans^1^, Roy F. Chemaly^2^, Burton F. Dickey^1^, and David E. Ost^1^

^1^Department of Pulmonary Medicine, The University of Texas MD Anderson Cancer Center, Houston, Texas, United States of America

^2^Department of Infectious Diseases, Infection Control and Employee Health, The University of Texas MD Anderson Cancer Center, Houston, Texas, United States of America

^3^Department of Diagnostic Radiology, The University of Texas MD Anderson Cancer Center, Houston, Texas, United States of America

^4^Department of Biostatistics, The University of Texas MD Anderson Cancer Center, Houston, Texas, United States of America

^#a^Current Address: Department of Epidemiology and Biostatistics, University of Texas Health Science Center at San Antonio, San Antonio, Texas, United States of America

**Address correspondence to:** Ajay Sheshadri, MD, MSCI. The University of Texas MD Anderson Cancer Center, email: [asheshadri@mdanderson.org](mailto:asheshadri@mdanderson.org)

**Supplemental Discussion**

**S1. Selection of Study Cohort When Using RSI to Predict Mortality.**

Our study population was ideal for testing RSI due to the high mortality of PIV-associated LRI in this population as well as the focus on early LRI. In our population of PIV-associated LRI, most subjects presented early in the course of LRI due to institutional policies to seek medical attention early in the course of any URI. As such, RSI scores at baseline were low in both survivors and non-survivors. Accordingly, more information could be gained by longitudinal assessment of RSI scores since the potential change (delta-RSI) from baseline to peak RSI was higher. This is reflected in our models showing the excellent discriminatory ability of delta-RSI for the prediction of 30-day mortality. Progression of RSI may be less informative in more severe cohorts, such as community acquired pneumonia (CAP) requiring direct admission to the ICU, since the baseline RSI is likely to be high and the additional information gained in the longitudinal assessment of RSI scores is lower. Conversely, in patients presenting with early LRI, assessing RSI longitudinally may have higher predictive value. However, the use of RSI in other populations of LRI requires validation.

The discordance of other studies (1-3) with regard to whether baseline radiologic severity, progression of radiologic severity, or delayed resolution of radiologic infiltrates predict mortality is likely multifactorial. Study populations vary in the baseline risk for death in subjects. For example, Mittl et al. enrolled a mixed population of 81 inpatient and outpatient subjects with community acquired pneumonia (CAP) of which only 1 patient died (1), while Bruns et al. enrolled patients with severe CAP at the time of hospitalization of which 6.9% died (2). The baseline risk for death for subjects enrolled in those two trials was likely to be lower patients enrolled in the study by Lisboa et al. (3), who were admitted to the ICU with severe CAP and of whom 27.7% died. We enrolled a high-risk cohort of immunocompromised patients with a historically high risk of mortality after PIV LRI and found that 14% died by day 28 and 17% died by day 60. Accordingly, both the studies by Lisboa et al. and our group both found that radiographic progression of pneumonia predicted mortality, whereas the studies by Bruns et al. and Mittl et al. of patients with a lower baseline risk for death did not show the same association. It is likely that we and Lisboa et al. found an association with LRI progression and mortality where Bruns et al. and Mittl et al. did not as the latter two studies had baseline mortality rates that were low relative to the size of the cohort, whereas we and Lisboa et al. studied populations with higher baseline mortality rates. Therefore, the baseline risk for death in a study population may in part determine the ability of progression of radiologic severity, as measured by RSI, to predict mortality.

Prior studies did not use the presence of pneumonia as a time-varying covariate in Cox models as we did. For example, Bruns et al. obtained imaging only at baseline, on day 7, and on day 28 and used logistic regression models to predict 28-day mortality (2). Mittl et al. used a Cox proportional hazards regression to model to identify risk factors for time to radiographic clearance of pneumonia, but not time to death since they had only one fatality (1). Lisboa et al. described four categorical patterns of radiographic progression or resolution and used logistic regression models to predict ICU mortality (3). However, due to large differences in mortality between groups, their use of logistic regression models was sufficiently powerful to show an increased mortality with rapid radiologic progression of pneumonia. Our use of RSI as a time-varying covariate gave us the ability to measure radiologic severity at a granular level longitudinally. In addition, the use of Cox methods allowed us to use all information in survivors and non-survivors up until the time of death or censoring and to overcome the measurement bias inherent in clinical practice, in which sicker patients have more frequent assessments of radiologic severity than do healthier patients, and patients who die are no longer available for assessment of radiologic severity. Finally, RSI quantifies severity as a continuous variable; therefore the use of RSI in longitudinal analyses allows for a more precise definition of changing severity than categorical patterns of radiographic progression and resolution as in the studies discussed here (1-3).

In conclusion, RSI may perform differently depending upon the chosen study population’s baseline risk of death and on the type of statistical analysis used (e.g. Cox vs. logistic regression). These are important considerations when planning to use RSI in prospective studies. In appropriate populations, RSI may be an excellent surrogate biomarker for mortality.

**References**

E1. Mittl RL, Jr., Schwab RJ, Duchin JS, Goin JE, Albeida SM, Miller WT. Radiographic resolution of community-acquired pneumonia. Am J Respir Crit Care Med. 1994;149(3 Pt 1):630-5.

E2. Bruns AH, Oosterheert JJ, Prokop M, Lammers JW, Hak E, Hoepelman AI. Patterns of resolution of chest radiograph abnormalities in adults hospitalized with severe community-acquired pneumonia. Clin Infect Dis. 2007;45(8):983-91.

E3. Lisboa T, Blot S, Waterer GW, Canalis E, de Mendoza D, Rodriguez A, et al. Radiologic progression of pulmonary infiltrates predicts a worse prognosis in severe community-acquired pneumonia than bacteremia. Chest. 2009;135(1):165-72.
