# Supplementary material for: Progression of the Radiologic Severity Index predicts mortality in patients with parainfluenza virus-associated lower respiratory infections
Source: PLoS One. 2018 May 17;13(5):e0197418. doi: 10.1371/journal.pone.0197418 (PMC5957350; doi:10.1371/journal.pone.0197418)
Supplement: S2 Discussion — This section offers a brief discussion on pitfalls of data sampling in retrospective and prospective cohort studies, with suggestions on optimal data sampling techniques for potential prospective studies using RSI as a biomarker of radiological pneumonia severity. (DOCX) [file pone.0197418.s002.docx]

**Progression of the Radiologic Severity Index Predicts Mortality in Patients with Parainfluenza Virus-Associated Lower Respiratory Infections (Online Supplement)**

Ajay Sheshadri^1*^, Dimpy P. Shah^2#a^, Myrna Godoy^3^, Jeremy J. Erasmus^3^, Juhee Song^4^, Liang Li^4^, Scott E. Evans^1^, Roy F. Chemaly^2^, Burton F. Dickey^1^, and David E. Ost^1^

^1^Department of Pulmonary Medicine, The University of Texas MD Anderson Cancer Center, Houston, Texas, United States of America

^2^Department of Infectious Diseases, Infection Control and Employee Health, The University of Texas MD Anderson Cancer Center, Houston, Texas, United States of America

^3^Department of Diagnostic Radiology, The University of Texas MD Anderson Cancer Center, Houston, Texas, United States of America

^4^Department of Biostatistics, The University of Texas MD Anderson Cancer Center, Houston, Texas, United States of America

^#a^Current Address: Department of Epidemiology and Biostatistics, University of Texas Health Science Center at San Antonio, San Antonio, Texas, United States of America

**Address correspondence to:** Ajay Sheshadri, MD, MSCI. The University of Texas MD Anderson Cancer Center, email: [asheshadri@mdanderson.org](mailto:asheshadri@mdanderson.org)

**Supplemental Discussion**

**S2. Measurement Bias and Data Sampling in Retrospective and Prospective Cohort Studies.**

We acknowledge that measurement bias may exist because sicker patients were likely to have more assessments, and patients who died may not have had radiologic assessment at peak severity. However, the lack of follow-up studies in healthier patients would likely bias our results towards the null hypothesis, since we do not capture resolution of pneumonia. Furthermore, the lack of radiology in patients who died before follow-up studies could be obtained would also bias our results towards the null hypothesis, because we would miss the progression of severe pneumonia. Finally, our method of imputation ensured that our model likely captured progression of clinical disease, since radiology is typically performed due to changes in clinical status and not performed when a patient’s clinical status is stable. The last observation carried forward (LOCF) method of imputation assumes that RSI remains the same between radiologic assessments and resembles the prior measurement more than it does the next subsequent measurement. This is a reasonable assumption because if a radiograph is not done, then it would be reasonable to assume that the patient’s clinical status has not changed enough to merit another radiologic evaluation. When clinical changes occur, chest radiographs are likely to be done in a timely manner, and therefore rapid changes are still likely to be detected by clinically driven radiographic studies. If patients have subtle changes in clinical status that occurred but did not result in a radiologic evaluation, our method of imputation would incorrectly reflect the true radiologic severity and either the score would be falsely low (if no radiograph was ever done) or it would lag behind the true change in time (if the radiograph was eventually done). In either event, this would bias our results towards the null hypothesis. However, despite these sources of measurement bias that would favor the null hypothesis, our study shows that RSI remains highly associated with mortality after PIV-associated RSI.

A prospective study employing RSI could obtain measurements at pre-specified points in time. However, such a strategy is not necessarily more efficient than radiologic assessments obtained driven by clinical judgment. It is best to think of this as a problem with data sampling. Because we are not able to measure every moment, how can we obtained unbiased and informative data efficiently? If a study only measured RSI at fixed points in time (e.g. days 1, 5, 10, and 21) and the outcome was at day 28, it is probable that the study would miss clinically significant changes in clinical status that occurred between those points in time, and imputation of RSI in those intervening points in time would not accurately reflect changes in radiologic severity. The illusion is that a fixed sampling schedule would eliminate the problem of imputation, but as long as the number of observations is fixed and equal to the number of observations taken by a clinical judgment strategy, fixed sampling does not provide more accurate data than clinically-driven sampling. If the frequency of assessments of RSI measurements is increased to a daily schedule, the problem of imputation is mitigated. But, that strategy is limited by concerns of radiation exposure and cost. The key is to recognize that for any given number of observations (in this example, four) a fixed schedule is actually inferior to a schedule of sampling which is driven by clinical judgment. A fixed schedule specifies a minimum number of observations per patient. However, a fixed schedule, on its own merits, does not have any benefit over a clinically-driven schedule of sampling – it is not nearly as good as clinical judgment because it is sampling in a semi-random manner which is not contingent on available patient data. Furthermore, validation of a score derived from radiologic testing performed on a fixed schedule may not be valid for clinical use, where testing is typically performed in response to clinical changes or persistent symptoms. However, the converse is not true – clinically-driven radiologic testing is likely to reflect clinically meaningful changes in patients, and the only additional value of a fixed schedule is to document resolution of LRI, stability of mild LRI, and potentially progression of severe LRI, where the reason for clinical deterioration is not in question. Therefore, fixed sampling is potentially less informative than a clinically-driven schedule of sampling. But adding a fixed schedule of sampling to a clinically driven schedule of sampling could add precision. Prospective studies should consider using a fixed schedule (e.g. days 1, 5, 10, 21) with further assessments obtained as clinically indicated by changes in clinical status. This would allow those studies to have more power than either a fixed schedule or clinically-driven schedule alone by more accurately capturing progression and resolution of pneumonia.

In conclusion, our study found that progression of RSI is highly associated with mortality. We found this association despite multiple sources of measurement bias which would bias our results towards the null hypothesis. The question of an ideal frequency of data sampling applies to both retrospective and prospective studies, and no strategy perfectly captures the ground truth radiological severity. Despite these concerns, changes in RSI, as captured in a real-world setting, accurately reflect changes in the hazard of death as measured by our extended Cox model.
